# Actions of the fall prevention protocol: mapping with the classification of nursing interventions

**DOI:** 10.1590/1518-8345.2394.2986

**Published:** 2017-12-21

**Authors:** Vanessa Cristina Alves, Weslen Carlos Junior de Freitas, Jeferson Silva Ramos, Samantha Rodrigues Garbis Chagas, Cissa Azevedo, Luciana Regina Ferreira da Mata

**Affiliations:** 1 Undergraduate student in Nursing, Universidade Federal de São João Del Rei, Divinópolis, MG, Brazil.; 2 Doctoral student, Escola de Enfermagem da Universidade Federal de Minas Gerais, Belo Horizonte, MG, Brazil. Scholarship holder at Coordenação de Aperfeiçoamento de Pessoal de Nível Superior (CAPES), Brazil.; 3 PhD, Adjunct Professor, Escola de Enfermagem da Universidade Federal de Minas Gerais, Belo Horizonte, MG, Brazil.

**Keywords:** Accidental Falls, Nursing Care, Nursing, Patient Safety, Standardized Nursing Terminology, Risk Management

## Abstract

**Objective:**

to analyze the correspondence between the actions contained in the fall prevention protocol of the Ministry of Health and the Nursing Interventions Classification (NIC) by a cross-mapping.

**Method:**

this is a descriptive study carried out in four stages: protocol survey, identification of NIC interventions related to nursing diagnosis, the risk of falls, cross-mapping, and validation of the mapping from the Delphi technique.

**Results:**

there were 51 actions identified in the protocol and 42 interventions in the NIC. Two rounds of mapping evaluation were carried out by the experts. There were 47 protocol actions corresponding to 25 NIC interventions. The NIC interventions that presented the highest correspondence with protocol actions were: fall prevention, environmental-safety control, and risk identification. Regarding the classification of similarity and comprehensiveness of the 47 actions of the protocol mapped, 44.7% were considered more detailed and specific than the NIC, 29.8% less specific than the NIC and 25.5% were classified as similar in significance to the NIC.

**Conclusion:**

most of the actions contained in the protocol are more specific and detailed, however, the NIC contemplates a greater diversity of interventions and may base a review of the protocol to increase actions related to falls prevention..

## Introduction

The fall is considered an event that causes the individual to end involuntarily on the ground or another low level, with or without injuries^1^. This event may be due to intrinsic factors such as physiological or pathological changes, psychological factors, and drug side effects; or extrinsic, related to the behavior and activities of individuals in the environment in which they live^2^. In the hospital environment, patients are in the process of reestablishing their health and they are considered more vulnerable. Consequently, the falls increase the hospitalization period and the cost of treatment, besides causing physical and psychological discomforts to the patient^3^.

In the United States, it is estimated that one-third of people over 65 years old experience at least one fall a year, with recurrence in half the cases. Approximately 10% of falls result in serious injuries such as fractures, soft tissue injuries and traumatic brain injuries, which require urgent care^3^. By 2015, in the United States, about 2.8 million falls were recorded and more than 800,000 of the cases required hospitalization. The estimate of medical costs related to this incident is R$ 34 billion per year^4^.

The National Agency of Sanitary Surveillance (ANVISA) published an incident bulletin and identified 9,423 failures in the care of different health facilities. Of them, 3,600 (38.2%) referred to the fall, is the second cause of notifications. The most common causes are a loss of balance, followed by slipping and syncope. The furniture also contributes, being the bed fall as the most reported, followed by falls in the bathroom and the chair^5^.

The likelihood of injury to health through accidents, illness, suffering or environmental factors is called risk^6^. Patient safety understood as interventions that minimize unnecessary harm to the care to an acceptable minimum, has become a worldwide concern since unsafe practices imply risks^7^.

Through Ordinance 529, on April 1, 2013, the Ministry of Health established the National Patient Safety Program (PNSP) to collaborate in the qualification of health care. In the PNSP, six protocols were described, including the fall prevention protocol, whose contents contemplate several actions with the intention of strengthening fall prevention strategies^8^.

Therefore, given the patient’s safety context, it is known that nursing is indispensable in the implementation of safe practices, since, through preventive interventions, nurses have the skills to make decisions regarding care to adequate and harmless assistance^3^ ^,^ ^9^.

To make nursing practices more effective, nursing classification systems, useful tools to guide the nurses’ clinical reasoning and establish standardized languages and, consequently, improve the care provided from the scientific base^9^. Regarding the Classification of Nursing Interventions (NIC), intervention is defined as any treatment based on the clinical judgment that the nurse performs to improve the human response to a health condition or life process experienced by a person, group or community. The NIC is comprised of 554 nursing interventions and approximately 13,000 activities, grouped into seven domains and 30 classes^10^.

However, it must be recognized that expanding the use of classification systems in clinical practice is a major challenge for nursing care. Thus, national and international studies have been developed based on the methodology of cross-mapping which allows linguistic and semantic comparison between non-standard terminologies and classification systems^11^ ^-^ ^13^. It should be emphasized that cross-mapping is the method enabling the insertion of the standardized nursing language in health institutions since it allows a consistent comparison between the practice already developed by the nurses and the content of the classification systems^14^.

Although the fall prevention protocol presents a multi-professional approach, most of its actions are performed by the nursing team, which remains longer direct care to the patient when compared to other health professionals. In view of the diversity of actions contained in this protocol, the need arises to compare it with the standardized nursing language to investigate the applicability of the NIC regarding the patient safety, specifically to the prevention of falls. The protocol is an objective tool, easy to access and free of charge. For this reason, it is believed that the results of this research can subsidize important advances in nursing care and highlight the importance of standardized language in helping to prevent falls.

In this context, the study had as objective to analyze the correspondence between the actions contained in the protocol of falls prevention of the Ministry of Health with the NIC by means of the cross-mapping.

## Method

This is a descriptive study performed by cross-mapping. It is a useful tool to analyze the data contained in the nursing process, comparing existing information with the reference classifications, as NIC in this case^15^.

Data collection was carried out between May and December 2016 and the research was carried out in four stages. The first step included the study of the fall prevention protocol of the Ministry of Health^16^ to identify and list preventive actions.

In the second stage, interventions for the prevention of falls in the NIC were selected through consultation with the NANDA-I/NIC link from the nursing diagnosis (ND) “risk of falls”^17^. All the interventions and priority suggested additional optional nursing activities to solve the problem were listed, according to their definitions.

The third stage consisted in the development of the cross-mapping between the actions of the protocol and NIC interventions related to the “risk of falls”. The instrument developed by the authors to develop the mapping contemplated the actions of the protocol organized in three thematic categories in the left column (direct practices for fall prevention, patient/family orientations, evaluation, and monitoring); and a column to the right where the corresponding NIC interventions were inserted, with the description of the domain, class, NIC intervention title and activities. The following rules^18^ were used and adapted to develop the mapping: list of the actions related to the “risk of falls” found in the protocol; list of the NIC interventions related to the ND “risk of falls” by NANDA-I/NIC link; map of the actions that consisted of linking each nursing care to a specific intervention according to NIC, using the context of the “risk of falls”; use more specific and appropriate NIC interventions; map meaning versus words, not words only; use the action keyword listed in the protocol to map the NIC intervention; ensure consistency between the definition of the intervention and the action to be linked; use the title of the more specific NIC intervention; map the NIC intervention based on its title and definition, considering the most appropriate activities; consider actions that have two or more verbs in distinct items, to become two or more corresponding actions.

The fourth stage corresponded to the analysis and refinement of the mapping by expert nurses, using the Delphi technique, a method used to obtain the consensus of opinions among a group of specialists through the application of structured questionnaires, circulating among the participants, with the statistical feedback of each response. Regarding the number of skilled nurses, this method does not establish the number of participants to guarantee the representativeness of the results but defines that success refers to the qualification of the participants^19^. Therefore, for the selection of the five experts, inclusion criteria were the minimum practical experience of five years, a doctorate degree in nursing and knowledge for the use of NIC. The instrument elaborated by the authors was constructed in columns, one containing the actions of the protocol of falls raised in the first stage of the study, another with the NIC interventions and their respective activities. The expert nurses were instructed to write notes alongside each activity (agree or disagree), and description suggestions, if necessary. If they disagreed, they should suggest NIC intervention to be mapped. In a third column, the experts also classified the mapping according to the similarity and comprehensiveness of the actions/interventions mapped: A- Identical terms - the nursing action of the protocol is identical in words and definitions to the activity proposed by the NIC; B- Similar terms - the nursing action of the protocol is comparable and similar in meaning to the activity proposed by the NIC; C- General and broad terms - the nursing action contained in the protocol is general and broad, that is, less specific in relation to NIC activity; D- Detailed and specific terms - the nursing action contained in the protocol is more detailed and specific when compared to NIC activity^20^.

For data analysis, Excel version 2016 was used to calculate the concordance of the mapping through the frequency analysis. The 80% index was adopted as the minimum level of agreement in the mapping validation^21^. The project was approved by the Research Ethics Committee of the proposing institution, under opinion 1,653,406, CAAE: 56911716.0.0000.5545, complying with Resolution 466/2012, which regulates research with human beings.

## Results

We identified 51 actions in the fall prevention protocol of the Ministry of Health, organized into three thematic categories: direct practices for falls prevention (n = 23); patient / family guidelines (n = 14); evaluation and monitoring (n = 14).

In the second step, the 42 NIC interventions related to the NANDA-I risk of falls were listed, being 19 of the basic physiological domain, 10 of the behavioral, eight of the safety domain and five of the physiological complex.

In the third stage, 25 (59.5%) of the 42 NIC interventions corresponded to 43 (84.3%) of the protocol. The NIC interventions with the highest correspondence with the protocol actions were: prevention of falls (6490) (n=26), control of the environment - safety (6486) (n=7) and risk identification.

The fourth stage of the study contemplated the analysis and refinement of the mapping by expert nurses, from two Delphi rounds. As for the characteristics of the experts, most were females (80%) with an average of 14 years of professional experience (Table 1).

**Table 1:** Characterization of the sample of expert nurses. Divinópolis, MG, Brasil, 2016

**Characterization of the sample**	**n**	**%**
**Women**	**4**	**80.0**
**Professional experience**		
**5 to 10 years old**	**1**	**20.0**
**10 to 20 years old**	**3**	**60.0**
**20 to 25 years old**	**1**	**20.0**
Scientific activities with the topic of Patient Safety and NIC *		
**Publication in magazines or periodicals**	**4**	**80.0**
**Presentation of work in scientific event**	**3**	**60.0**
**Subject in master’s dissertation or doctoral thesis**	**1**	**20.0**

* NIC - Nursing Interventions Classification.

In the first round, the experts analyzed the matching of 43 protocol actions with 25 NIC interventions. The level of agreement above 80.0% was obtained in 88.4% (n=38) of the actions. Four actions of the protocol considered as unmapped (assessing the level of dependence and autonomy after the installation of equipment, guiding the patient to get up progressively from the bed and with the help of a professional of the care team; and reported on the occurrence of falls) were considered to be mapped by some experts and therefore included in the second round of Delphi together with the five mapped protocol actions that obtained agreement below 80.0% (periodically review and adjust prescription of medicines that increase the risk of falls, stimulate the preparation and distribution of educational material to prevent the risk of falls, guide the person responsible for the influence of the diagnosis on the increased risk of falling, identify the patient at risk by means of Bedside or bracelet, and periodically supervise comfort and safety of the patient).

The result of the second round Delphi pointed out that 90.7% (n = 39) of the cross-mapping obtained agreement of 80%, and 9.3% (n=4) agreement of 100% among experts. Thus, the final mapping presented correspondence of 25 (59.5%) NIC interventions with 47 (92.2%) protocol actions. It should be emphasized that there were actions of the protocol that corresponded with more than one NIC intervention (Table 2).

**Table 2:** Mapped NIC interventions and a corresponding number of protocol actions. Divinópolis, MG, Brazil, 2016

NIC Intervention Code *	NIC Title Intervention *	**Number of mapped actions n (%)**
	**Priorities**	
**6490**	**Preventing falls**	**26 (55.3)**
**6486**	**Control of the environment: safety**	**7 (14.9)**
	**Suggested**	
**6610**	**Identification of risk**	**7 (14.9)**
**0970**	**Transfer**	**6 (12.8)**
**1806**	**Self-care assistance: transfer**	**5 (10.6)**
**2380**	**Medication control**	**5 (10.6)**
**1804**	**Self-care assistance: use of the toilet**	**3 (6.4)**
**5648**	**Teaching: infant safety 10-12 months**	**3 (6.4)**
**5665**	**Teaching: child safety 13-18 months**	**3 (6.4)**
**6460**	**Dementia control**	**2 (4.2)**
**2690**	**Precautions against seizures**	**1 (2.1)**
**0840**	**Positioning**	**1 (2.1)**
**0846**	**Positioning: wheelchair**	**1 (2.1)**
**0590**	**Control of urinary elimination**	**1 (2.1)**
**5645**	**Teaching: infant safety from 0-3 months**	**1 (2.1)**
**5646**	**Teaching: infant safety for 4-6 months**	**1 (2.1)**
**5647**	**Teaching: Infant Safety 7-9 Months**	**1 (2.1)**
**5666**	**Teaching: child safety 19-24 months**	**1 (2.1)**
**5667**	**Teaching: child safety 25-36 months**	**1 (2.1)**
	**Optional**	
**6440**	**Control of Delirium**	**1 (2.1)**
**1800**	**Self-Care**	**1 (2.1)**
**4974**	**Communication improvement: Hearing impairment**	**1 (2.1)**
**0221**	**Exercise Therapy: Ambulation**	**1 (2.1)**
**0430**	**Intestinal control**	**1 (2.1)**
**2130**	**Control of hypoglycemia**	**1 (2.1)**

* NIC - Nursing Interventions Classification.

**Figure 1:**
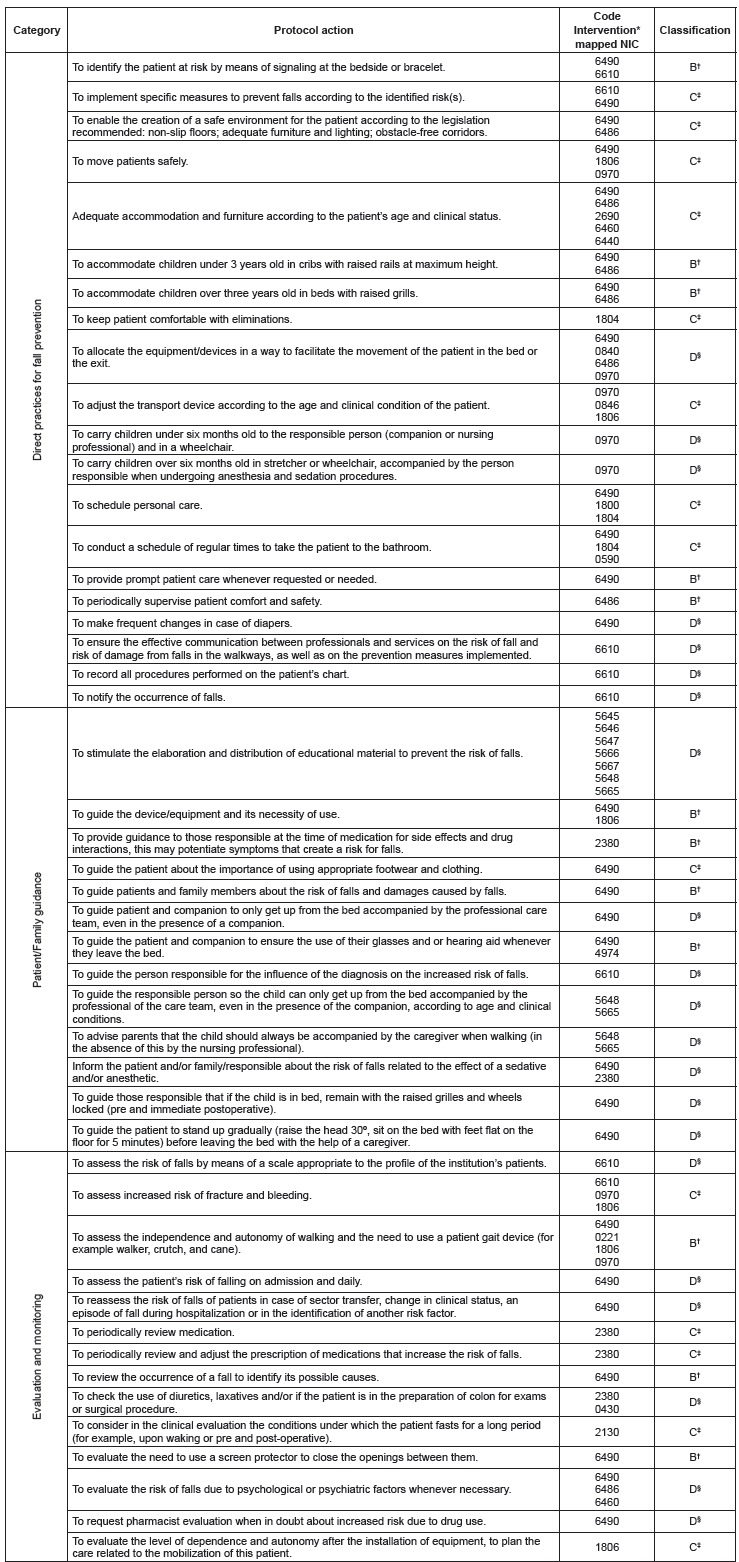
Cross-mapping of the actions of the fall prevention protocol with NIC after expert analysis. Divinópolis, MG, Brazil, 2016

Regarding the classification of similarity and comprehensiveness of the 47 actions of the protocol mapped^20^, 44.7% of the actions of the protocol were considered more detailed and specific than the NIC, 29.8% less specific than the NIC and 25.5 % were classified as similar in significance to the NIC (Figure 1). It should be noted that no action of the protocol was classified as identical to a NIC intervention/activity.

*NIC - Nursing Interventions Classification. †B - Similar Terms; ‡C - General and broad terms; §D - Detailed and specific terms.

**Figure 2:**
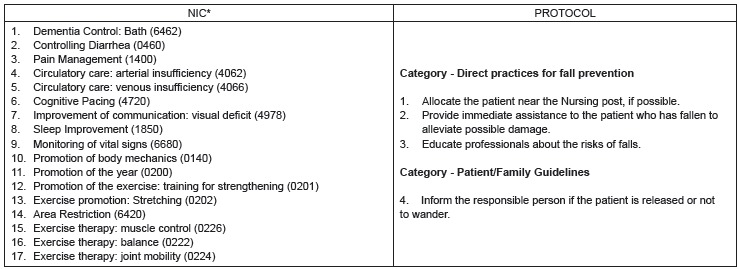
NIC interventions and unmapped protocol actions. Divinópolis, MG, Brazil, 2016

Figure 2 shows the 17 NIC interventions and the four actions of the protocol that did not correspond, after analyzing the experts.

*NIC - Nursing Interventions Classification

## Discussion

Regarding the main results of the first stage of the study, which refers to the survey of 51 actions of the protocol of the Ministry of Health for the prevention of falls, it is noted the prioritization of care related to “direct practices for prevention”, such as: identify the patient at high risk through signaling at the edge of the bed or bracelet, move the patients safely, allocate the patient at high risk for falls near the nursing station and arrange personal hygiene care. On the other hand, an international study, whose objective was to identify the most effective actions to prevent falls in adults, identified the valuation of practices focused on environmental control. The most applied activities in the hospital sectors were to maintain high bed grids, to wear non-slip shoes and to keep the call light within reach of the patients^22^.

The protocol also highlights the importance of involving the patient and the family in the prevention of falls, since it contemplated 11 actions in this context. Among the most used strategies, it is mentioned the education of the patient/companion, mainly in Brazilian reality, in which the hospitalized patient, in most cases, has the presence of the companion^23^. Actions such as guiding not to get up from the bed alone and warn about the risk of falls related to the use of sedative, anesthetic, and medical diagnosis, are important, as they encourage patients and caregivers to take a proactive role in caring. On the other hand, a Brazilian study characterized the falls of patients in a cardiology unit, identified that 50% of them were followed up, that is, the presence of the companion did not prevent the occurrence of this adverse event^24^. Therefore, it is important to emphasize the role of the nursing team in the adoption of diversified strategies for the education of patients and family/caregivers, because to reduce the incidence of falls, they must truly understand the risk factors as well as their real responsibilities for prevention of falls.

Interventions involving “evaluation and monitoring” are also of great relevance for fall prevention in the context of the Ministry of Health protocol. Assessing the risk for falls is an essential component of any prevention program to identify patients at risk to correct the situation and, finally, to avoid the occurrence of falls. It is recommended that the evaluation is performed at the admission of the patient and at least every three days during the period of hospitalization and when there is a transfer of the unit, there is a change in its clinical condition or after the occurrence of a fall^25^. The evaluation has been based on fall risk assessment scales, tools that assign numerical values to several factors, and the sum of these predicts whether the patient has a risk of low, medium or high falls^23^.

In general, the assessment for the risk of falls involves the collection of factors such as history of falls, mental and sensory alteration, mobility, age, medications in use, the presence of osteoarticular diseases, alterations of balance, inactivity, and alterations of vision and hearing. The identification and evaluation of these factors in an individualized way allows the implementation of fall prevention strategies according to the characteristics presented by the patient^25^.

Regarding the possible contributions of the risk assessment for falls, a Japanese study described the effectiveness of a prevention program and identified a 60% reduction in the drop rate of hospitalized patients. This multidisciplinary program was based on interventions that included assessment of the risk of falls through a standardized instrument, modifications to the environment, teaching to the patient, family/companion and team, and implementation of a prevention protocol in which patients with at least one were considered to be at high risk. Fallout educational material was provided to patients and caregivers, including posters alongside the beds, and for patients considered unable to request assistance, movement alert devices were used^26^. In the context of these actions, identifying patients at high risk for falls is a relevant nursing assignment, so that individualized and specific interventions are implemented for each patient according to the risk factors presented.

Despite the existence of different instruments for the assessment of the risk of falls, it is important that the health services use those that are validated for the specific populations to minimize the chances of bias or error in the identification/classification of the risk^25^. Among the instruments for this purpose, there is the Morse Fall Scale (MFS), one of the most studied at an international level, applied in several scenarios mainly in adult patients. This is the first instrument to be directed to the elaboration of a care plan based on specific nursing interventions, aimed at overcoming the risk of falling^27^. It is important to consider that the MFS was submitted to the cross-cultural adaptation process for use in Brazil,^23^ However, there is no publication that references the accuracy analysis of the Brazilian version, which highlights the importance and necessity of new studies.

In Brazil, the St Thomas Risk Assessment Tool in Falling Elderly Inpatients (STRATIFY), developed in England in 1997, has been translated and adapted with the inclusion of some items, such as medication use and age equal or superior to 60 years old. However, there are no Brazilian studies about its validation^16^ ^,^ ^28^. There is also the Johns Hopkins Fall Risk Assessment Tool, which has undergone translation, cross-cultural adaptation, and content validation, with satisfactory results for the evaluation of the risk of falls in the Brazilian population^29^.

Concerning the main results of cross-mapping, 47 of the 51 actions contained in the protocol for falls prevention were mapped to 25 NIC interventions. The NIC interventions that presented the highest correspondence with the actions of the protocol were prevention against falls (6490), environmental control (6486) and risk identification (6610). These findings corroborate the results of a study that identified nursing care prescribed for hospitalized patients at risk of falls. The most frequent NIC interventions were control of the safety environment (6486) and prevention of falls (6490), differing only in relation to intervention risk identification (6610), which was not mentioned^30^. It should be emphasized that the three interventions that presented the highest correspondence in this study are classified as priorities in the NIC, that is, they are the most probable for the resolution of ND “risk of falls”^10^.

The intervention NIC drug control (2380) was mapped four times. It is known that some medications may contribute to the occurrence of falls, especially in the elderly and hospitalized patients. The drug classes that are most associated with falls are hypoglycemic, antihypertensive, psychotropic and opioid^31^. A study whose objective was to evaluate whether patients who had fallen did use some medication, indicated that 95.4% of the patients used at least one drug associated with falls^32^. The use of drugs associated with the risk of falls reinforces the idea that all health staff, especially nursing, should take an active role in drug control in order to identify patients at high risk.

Among the non-mapped NIC interventions there are the promotion of body mechanics (0140), promotion of stretching exercise (0202) and exercise therapy: muscle control (0226), exercise therapy: balance (0222), exercise therapy: joint mobility). The non-identification of these interventions in the protocol signals the importance of a review and inclusion of actions related to muscle stimulation since one of the main risk factors for falls is the reduction of muscle strength. An international review has shown that increased muscle strength results in a lower risk of falls and injuries, especially in the elderly. Muscle weakness is a factor strongly associated with the risk of falls, especially in certain situations, such as slipping when taking a step and falling when trying to get up from the bed or chair. Muscle strengthening exercises such as stretching, increased balance, endurance, and flexibility significantly reduce the risk of falls^33^.

The pain control NIC intervention (1400) also did not correspond to the actions present in the protocol. However, pain is one of the most prevalent symptoms in people over 65 years old and can cause physical restrictions and changes in the level of consciousness, being a relevant risk factor for falls^34^. A systematic review proposed to identify an association between pain level and risk of falls in the elderly and found that pain was the most significant factor in relation to cognitive capacity, the presence of depression, visual impairment and use of sedatives^35^. These results suggest the importance of inserting actions in the protocol regarding pain monitoring and control, such as periodically assessing pain levels, ensuring pharmacological and non-pharmacological care, and reducing factors that cause pain, mainly considering the occurrence of previous falls, which aims to reduce this incident.

The NIC interventions monitoring vital signs (6680), circulatory insufficiency care (4062), circulatory insufficiency venous insufficiency (4066) and cognitive stimulation (4720) were also not mapped. The American Society of Geriatrics points out that postural hypotension is associated with the risk of falls. Supervising patients on the use of hypotensive drugs, preventing dehydration, stimulating the use of elastic stockings in patients with indication should be practical for the prevention of hypotension and, consequently, of falls^36^. Regarding cognitive stimulation, inadequate blood pressure values are associated with cognitive impairment, which increases the risk of falls threefold. Therefore, cognitive functioning may serve as an intermediate mechanism between changes in blood pressure and the risk of falls, especially in the elderly, that is, the probability of falling is highly evidenced^37^.

Another unmapped NIC intervention was sleep improvement (1850). Sleep deprivation is an influential point for the risk of falls in the elderly, as it can cause daytime sleepiness, cognitive dysfunction and reduction in reflex response time^38^.

The NIC intervention improved visual communication deficit (4978) also did not match the protocol. The association of visual dysfunction with the risk of falls is significant since hospitalized patients with decreased visual acuity have a higher frequency of falls. Physiological changes in the eyes resulting from aging include gradual loss of visual acuity, decreased peripheral vision, visual accommodation, the perception of depth, and slowness in the process of visual information. The visual system plays an important role in postural maintenance and alterations may impair the maintenance of balance^39^. It is important that the protocol addresses the visual deficit, in order to propose actions that may reduce the risk of falls.

Four actions of the fall prevention protocol did not correspond to the NIC: to educate professionals about the risks of falls, and inform the responsible person if the patient is released or not to walk; allocate the patient near the nursing station, if possible; and provide immediate assistance to the patient who has fallen to alleviate possible damage. Unattended falls are more likely to result in injuries with serious consequences for the patient, which reinforces the importance of constant vigilance of patients at high risk^40^. Educating the professionals about the factors related to the occurrence of falls is a way to demonstrate the importance of this in the control of falls, besides encouraging the understanding that the risk assessment must be linked to prevention interventions, with a view to patient safety.

Sharing responsibility for prevention benefits the patients, the professionals and the institution^41^. Actions such as informing the patient and the patient about their release for ambulation or not, the risk of orthostatic hypotension, anesthesia effect, and prolonged fasting are fundamental to avoid early ambulation and consequently falls, especially in the postoperative period^28^.

It is suggested that the action of the protocol “to provide immediate assistance to the patient who suffered a fall to mitigate the possible damages” did not correspond to the NIC since it does not refer to a prevention practice, but to act through the incident.

Australian study reinforces the importance of professional training. The researchers investigated the impact of an online training program for nurses on fall risk assessment, post-fall prevention and management, and identified that the proposal contributed significantly to the implementation of interventions such as: introducing warnings about the risk of falls in the patient charts during mobilization or in the bathroom, elimination of risks present in the environment, use of alarms in the beds and in the chairs, and referral to other health professionals. The findings enabled to conclude that educational programs, aimed at professionals, represent a positive cost-effective method for the improvement of fall mitigation strategies in health organizations^42^.

Regarding the limitations of the study, it is suggested the possibility of not having contemplated all the actions contained in the protocol, due to its narrative structure. Successive readings of the protocol were carried out to list all actions, and, therefore, it is believed that the methodological approach, including the validation of the mapping by experts, contributed to a greater reliability of the results.

## Conclusion

The cross-mapping allowed the comparison of the actions contained in the protocol of falls prevention of the Ministry of Health with the interventions standardized by the NIC. Of the 51 actions contained in the fall prevention protocol, 47 were mapped to 25 CIN interventions. It was also found that four (7.8%) protocol actions were not mapped, along with 17 NIC interventions (40.5%).

The validation of the mapping showed that the actions contained in the fall prevention protocol were considered more specific and detailed. However, the NIC contemplates a greater number of interventions, enabling to conclude that the protocol is capable of expanding new interventions with a view to reducing the risk of falls, including, for example, improvement of sleep, improvement of communication: visual deficit and pain control. It was observed that the majority of non-mapped NIC interventions are related to the stimulation of muscle strength, which evidences the need to include such interventions since muscle limitations are important risk factors for falls.

Finally, it is recommended that unmapped NIC interventions be integrated into the protocol, as well as protocol actions that have not been mapped to be proposed for NICs since such interventions and actions can contribute to the prevention of falls to improve quality and safety in healthcare.
